# Meeting of the American International Congress on Tuberculosis, Held under the Auspices of the Universal Exposition of St. Louis, October 3, 4 and 5, 1904

**Published:** 1904-11

**Authors:** 


### Meeting of the American International Congress on Tuberculosis, Held Under the Auspices of the Universal Exposition of St. Louis, October 3, 4 and 5, 1904.

The Congress was a most gratifying success. Influences were set in motion that doubtless will result in great good. The attendance was large and constant; more than could have been expected, and the interest and enthusiasm were very marked. I say more than could have been expected, because of the simultaneous occurrence of so many interesting things to attract and divert delegates—the Fair itself, the Veiled Prophet festival, the New York ball, Missouri day and reception, the presence of the governors of several States, who received ovations, the simultaneous session of the military surgeons, and other congresses, etc. I give below a synopsis of the proceedings compiled from notes of the Secretary and from the *Texas Medical News.* The whole will be published in the *Medico Legal Journal* and the *Bulletin of the Congress,* by Judge Clark Bell, 39 Broadway, New York.

FIRST DAY, OCTOBER 3.

President Dr. E. J. Barrick, of Canada, in the chair.

The address of welcome was delivered by Col. Pat Dyer, United States District Judge, on behalf of the United States government.

Dr. Chas. H. Hughes, of St. Louis, was next introduced, and welcomed the Convention to the city of St. Louis, and wished them godspeed in their great work. He referred to the great death rate of the disease, and the feeble efforts made as a rule to resist the spread of such a preventable disease. He urged the adoption of resolutions of this Association demanding the passage of laws for the protection of the public against consumption in its various forms.

Governor L. F. C. Garvin, of Rhode Island, was then introduced, and spoke of the prevalence of the disease, and referred to the active work now being done in his State, not only for the treatment and isolation of the afflicted by the recent building of a State sanitarium for such cases, but active efforts were being made all over the State to prevent the spread of the disease, and in closing referred in an eloquent manner to the necessity for all States as well as the National government to take active steps for the care and treatment of their tubercular people.

Dr. A. N. Bell, of Brooklyn, N. Y., the oldest sanitarian in the Association, was unable to be present, owing to his feebleness, being 84 years old. He was expected to make some remarks, but sent a paper, which, by motion, was read by Dr. J. H. Kellogg, of Battle Creek, Mich., and was received with great favor.

Hon. Clark Bell, of New York, was next introduced as the President of the Medico-Legal Society. He referred in an eloquent manner to the magnitude of the work of this Association, the assistance and co-operation given its officers by the United States government, by the Hon. John Hay, Secretary of State, and the officials of the Louisiana Purchase Exposition, as well as the governors and medical associations of the respective States. He also referred to the importance of having an association of this character, whose membership is not confined to the medical profession, taking in the legal profession, public officials, and, in fact, every one interested and able to assist in the prevention of this fearful disease. He also gave the history of the organization of this Association by the Medico-Legal Society.

Dr. E. J. Barrick, of Toronto, Canada, President of the Association, was next introduced and thanked the different speakers for their hospitable welcome to the city of St. Louis and its greatest exposition known to the world's history, and then delivered a most eloquent address* as President of the Association upon the great questions which must be discussed during the meeting.

In the absence of the Secretary of the Association, Dr. M. M. Smith, of Austin, Texas, was selected to act as Secretary for the Association.

Delegates from the various foreign countries were invited to have seats on the rostrum. Among those who responded on behalf of delegations, speaking at some length, were Drs. Kitchen, Morehouse, LeCavalier, and De Witt, of Canada.

Dr. Jacobsen, of Havana, Cuba, was next introduced, and spoke concerning the anti-tubercular league of Cuba, and referred to the work being done in his country concerning consumption.

Dr. Matto, of Peru, was next introduced, and spoke eloquently of the great interest taken in this humane work in his country, and what they hope to do in the way of sanatorial and climatic treatment of the disease.

Dr. C. T. Paloma, of San Salvador, then spoke at some length concerning the work being done in his country, and thanked the Association most courteously for their invitation to his government to officially attend this meeting.

Delegates from the various States of the United States were next called upon and introduced to the Association. Those speaking at some length before the Association were Dr. Anna C. Lyle, of San Francisco, Cal., who spoke feelingly concerning the great influx of consumptives to California from all portions of the United States, saying that most of their noted resorts had become infected foci, and that the natives were almost afraid to live in these places as a result. After speaking at some length concerning the little efforts made to prevent the spread of the disease in hotels, insane asylums, and other public places, she closed by urging this Association to pass strong resolutions outlining the duties of municipalities and States concerning indigent consumptives.

Dr. N. K. Foster, of Sacramento, Cal., Secretary of the Board of Health of California, referred to the recent organization of the anti-tubercular league of that State, and said that the schools of the large cities were now inspected by physicians.

Dr. P. O. Hansford, of Colorado Springs, Colo., said he believed the best results were obtained by placing all cases directly under police supervision, and favored the establishment of a school for the instruction of health officers.

Dr. M. M. Saliba, of Savannah, Ga., next spoke of the consumptives in his State, and said that formerly their cases were sent out of their State for treatment, if able, but they were now realizing that good results might be obtained at home if the patients were placed in the right surroundings.

---

*This and all the other addresses will appear in full in the Transactions.

Dr. Denslow Lewis, of Chicago, Ill., stated that active steps were now being taken in that State for the prevention of the disease; that a symposium upon the subject was presented at the last meeting of the Illinois State Medical Association. Since that time the profession and others were aroused to the situation.

Dr. Hinze-Scott, of Des Moines, Iowa, a delegate from the woman's league of her State, spoke of the great work being done by their organization for the education particularly of the mother, and through her, education of the children, as well as their proper development, as a means of preventing the disease.

Dr. Quitman Kohnke, President of the Board of Health of the city of New Orleans, spoke of the reduction of the number of cases in that city on account of their mild climate and the large number of hours spent in the open air by their people; said all cases were reported to the health officer, and the State was taking active measures to establish a sanitarium. He favored above all things as a means of preventing the disease proper and free ventilation of all houses.

Dr. J. H. Kellogg, of Battle Creek, Mich., advised the appointment of a committee to carry out the suggestions and resolutions made at this meeting, believing that committees should be appointed from this Association to report on the best methods of constructing sanitariums in the different climates, the best form of diet lists to be used by such patients, likewise outline rules concerning the habits, exercises, etc., for the patients, and likewise members of their families.

Dr. W. F. Morrow, of Kansas City, Mo., Secretary of the State Board of Health, spoke at length concerning the needs of education as the best preventive measure. He thought active work should begin in the State boards of health, and through them reach their respective counties in the State.

Dr. McAlister, President of the Board of Health of Missouri, also spoke concerning the active work being done in the State of Missouri to prevent consumption.

Dr. M. M. Smith, of Austin, Texas, Secretary of the Board of Medical Examiners for that State, was called upon by the President. He said that this is the most important meeting which would be held at the Louisiana Purchase Exposition. He agreed with Judge Dyer in his address of welcome when he said, if a State or a government has the authority to spend money and sacrifice lives for its protection from a foreign enemy, how much more important it would be to at least have the same right to appropriate money to prevent a disease within its boundaries which destroyed thousands. He believed one of the most important things to be considered was the education of the physicians all over the country, so that they would be able to diagnose a case in its very beginning, so that the patient could receive the best treatment, and the members of the family could be notified and prevent infection of others. He believed one of the greatest means of infection was the neglect of the family physician in diagnosing his case early and speaking plainly to the patient as well as the family, and urge them to combine their efforts, not only to prevent the disease in others, but to successfully restore to health the patient himself.

Dr. Robert F. Monahan, of Green Bay, Wis., referred to the infection of the disease and the small percentage of cures when neglected.

Many other short addresses were made.

SECOND DAY, OCTOBER 4.

The meeting was called to order by Dr. E. J. Barrick, President of the Congress, and Judge Clark Bell, of the Medico-Legal Society of New York, was introduced. His paper appeared on the program. He stated that, in order to facilitate matters, the paper might be read by title and published in the proceedings, which motion was adopted. The subject of the paper being "Preventive Legislation in Forensic Medicine." Judge Bell stated to the Convention that this was a time set apart for a symposium on Preventive Legislation on the Subject of Tuberculosis, and further stated that the question would be discussed as per the following resolution:

"Conceding that tuberculosis is a communicable disease from one human being to another, without which no legislation could be sustained by the courts, the real burning issues that confront the Congress may be thus briefly stated: (1) How far can legislation be devised that can arrest, avert, or even diminish, the terrible mortality of consumption, under which the human race now suffers? (2) How can this Congress devise means that will educate the public mind to a recognition of the imperative necessity of legislative action, and define its scope and field? and (3) to favor the passage of such legislation as is deemed likely to best accomplish the desired results; and (4) how can public opinion be best aroused, formed, and enlightened so that the public will favor the enforcement of such legislation when adopted?"

Dr. W. D. Neal, of Chicago, Ill., believed in the free discussion of the resolution as presented, and at once launched into a discussion of the communicability of tuberculosis, and said he did not believe in the dangers of the transmission of the disease as advocated by a majority of the members of the Congress, and as held by the medical profession of the world.

Dr. F. E. Daniel, of Texas, spoke as follows:

I have been of the opinion that the dictum of the immortal Koch as to the cause of consumption was universally accepted. I did not know that there was an intelligent and properly informed physician in the world who questioned the now generally accepted fact that the tuberculosis bacillus is the cause of the disease. I thought the etiology of the disease was now understood and accepted, and that, like the doctrine of evolution, it was the starting point for further research. I am, therefore, surprised that there should be offered here today papers that call it in question, and which, if read, would open up a controversy practically settled, and lead to an endless discussion. The censors appointed by the executive council of the Congress have very properly voted to exclude those papers. It would be profitless to thrash over the old straw and a waste of time to enter upon an academic discussion of the disease. We are not here to split hairs on clinical features and pathological conditions. The days for talking are past, and the time for action has come. We are here to *do something* for the arrest of the spread of that most insidious and deadly of all diseases, tuberculosis. This Congress was called for a definite and specific purpose. That purpose was announced broadcast over the world, and it is surprising that there are those here who do not understand what it was, and who would take up the limited time in a wrangle over points disputed, so far as I know, by no one but themselves. That purpose was to put in motion, if possible, influences that will awaken a public sentiment, and lead to legislation; to induce Congress and the States to put into operation measures to lessen the fearful mortality of consumption; to prevent its spread from the sick to the well, and ultimately to do what may be done toward the eradication of the disease.

We do not know all about consumption, but we know how it is caused, how propagated, how communicated from the sick to the well, and we know that its spread is preventable and it can be prevented by comparatively simple measures. But it requires authority and means to devise and enforce those measures, and our lawmakers alone can give that authority. We are here, then, for that purpose: to interest the public and the law-making bodies, to show them these things, to bring them to a realizing sense of the useless and perpetual waste of life by a preventable disease; in short, to set in motion influences that will lead to efficient action by the Federal Government and the States.

According to the United States census for 1900, there were that year 111,000 deaths from consumption in the United States in the registration districts alone. Those districts embrace about two-thirds of the population. Figuring on that basis, we find that there are, in round numbers, 150,000 deaths from consumption in America every year;—a number equal in five years to the entire losses on both sides, from all causes, during the four years' Civil war,—750,000. In ten years this means a million and a half of people lost by one. (preventable) disease alone. These figures are appalling, yet nobody except a few physicians and sanitarians seem to know it or to care, and these few have again and again asked in vain for legislation to stop it.

If America were invaded by an armed host, and our people,—men, women, and children,—were being slaughtered at the rate of over four hundred a day, every day,—every year,—all the time,—what would be the state of public sentiment and the attitude of government? Every recourse of science, skill, authority, action, and money would be brought into play; every energy and power exercised to arrest it; but, both the governments and the people are strangely apathetic in the face of the fearful ravages of the white plague. It is not indifference; it is because they do not know it and realize it, and know that there are means of arresting the ravages of the disease, its communication from the sick to the well, and that the predisposition to the disease can be greatly lessened by hygienic life and sanitary surroundings. They must be enlightened, as far as it is possible to enlighten them, for it is impossible to reach and impress the millions of unfortunates,—the under stratum of our social fabric,—the toilers who, in crowds, eat, sleep, and work in unsanitary houses and are poorly fed. If they could be reached and aroused they could not act. They are as children, and the government, in *loco parentis,* must act for them.

Mr. Chairman, there is interest, nay, enthusiasm, in this body, but, judging from what I have heard and the number of resolutions that have been introduced, there seems to be lack of understanding as to the direction that energy and enthusiasm should take. I suggest, sir, that a committee on resolutions be appointed to consider the several resolutions that have been read and to report upon them, setting forth the sense of this Congress as to the duties of Congress and the States in the premises; or, that this body memorial Congress to take immediate action to protect the public from the *tubercular infection.*

The motion prevailed, and the chair appointed: Dr. F. E. Daniel, Texas, Chairman; Dr. J. H. Kellogg, of Michigan; Dr. C. H. Hughes, of Missouri; Dr. Morrow, of Missouri; Dr. Denslow Lewis, of Illinois; Dr. Kohnke, of Louisiana; Dr. Hanchett, of Iowa; Dr. Kitchen, of Canada, and Dr. Foster, of California.

A paper was next read by Dr. Joaquin L. Jacobsen, of Havana, Cuba, president of the Anti-Tubercular League of Cuba. The subject was, "The Importance of Statistics in the Problem of Tuberculosis."

AFTERNOON SESSION.

At this session the symposium on the "Relation of Insanity to Tuberculosis" was declared to be open, and the secretary was requested to read the resolution which should form a basis for discussion for the Congress, which was as follows:

Whereas, Tuberculosis is directly or indirectly responsible for one-fifth to one-eighth of all of the deaths the world over, and for nearly one-half of those occurring between the ages of twenty and thirty years; and

Whereas, It is possible for each municipality to establish and maintain a municipal sanatorium for its own people, by bringing out the co-operation of Federal, State, provincial, municipal and individual aid; be it, therefore, and it is hereby

Resolved, That in the opinion of this International Congress on Tuberculosis all of the members thereof should use their influence in their respective localities to bring about this co-operation, which, if carried on in conjunction with the local boards of health, would bring the benefits of a sanatorium within the reach of consumptives in every municipality."

The President of the Congress, Dr. E. J. Barrick, rose and spoke at some length upon the importance of a free discussion of this important subject before the Congress. He referred to the admirable system of municipal sanatoria now being erected throughout Canada, and to the necessity of having ample provision made for the isolation of the tubercular in the insane asylums, jails, and penitentiaries, and urged that this important subject be brought forcibly to the attention of those in authority in such institutions. Motion was made that the papers relating to this subject, and presented by Drs. Hutchins, Morris, Benedikt, and R. J. Smith, and others, be read by title, and form a part of the proceedings of the Congress, after having passed the board on censorship as being suitable for publication. (This is a rule that is applicable to all papers presented to the Congress.) Motion was also made that the time for receipt of papers be extended to January 1, 1905, and that same should be considered to become a part of the proceedings of the Congress.

Dr. De Witt, of Nova Scotia, next addressed the Convention, and stated that he believed the great cause of tuberculosis was house infection; particularly was this the case in insane asylums. He stated that in his country the establishment of training schools for nurses in asylums had been the means of preventing the spread of the disease, through the efficient methods of disinfection as the result of experienced nurses in such cases.

Dr. Hays, superintendent of the insane asylum of Louisiana, said that in his State a new building was being erected for the isolation of the tubercular insane, and that on account of the mild climate in that State, and the large number of hours spent in the open air as a result, that they did not have the amount of tuberculosis among their insane that obtained in cooler climates and in less desirable atmospheres.

Dr. N. K. Foster, secretary of the State Board of Health of California, advocated the tent system for the isolation and treatment of tubercular insane, and believed the tent offered the best system of ventilation, and was cheap in construction. That it had been demonstrated throughout the West that it could be used in the winters as well as the summer with advantage, and he thought the benefits derived by their use would be very great.

Dr. M. M. Smith, of Texas, spoke of the isolation of the insane as carried on in the asylums in his State at the present time. He referred with pleasure to the fact that the isolation cottages for the tubercular had been made use of at one of the State penitentiaries of Texas for many years. And that the pioneer in this line of work was Dr. W. E. Fowler, resident physician of the State penitentiary at Rusk, Texas. Motion was made and adopted that a further discussion of this symposium should be postponed and kept open until January 1st. Said motion prevailed.

The next symposium open for the attention of the Convention was upon the "Pathology and Bacteriology of Tuberculosis."

A paper from Dr. Otto Von Schoen, of the Royal University of Naples, was presented to the Association in German and also in Italian, with the following titles: In Italian, "Sul Nuovo Microbe, della Tisi sulla differenza, essensiale Tra Tuberculosi e Tisi Pulmonale"; also one in the German language entitled, "Ueber den Phthisrogenen Microben, und den Unterschied zwishen Tuberculose und Phthise der Lunge." Motion was made that these papers be read by title and become a part of the transactions, but owing to the fact that they were in foreign languages, Dr. Frederick Kolbenheyer, of St. Louis, was introduced by the Hon. Clark Bell, and was asked to give the English synopsis of the German paper of Professor Von Schoen, which he did in a very interesting way, and referred to the great work and new discoveries made by this distinguished professor along the line of causation of various types of disease. After a free discussion of papers, the President submitted the names of the nominating committee to elect officers for the ensuing year.

Dr. P. O. Hanford, of Colorado, next discussed the paper, followed by Dr. Hare, of Colorado. Same was also discussed by Dr. Jaffa, of Colorado, and Dr. Finarty, of Iowa.

Professor C. H. Hughes, of St. Louis, delivered an eloquent discussion dealing principally with the value of *hope* in the treatment of tuberculosis. He referred to it as one of the greatest elements or aids in the successful treatment of a case. He spoke particularly of the mental side of the treatment of tuberculosis.

Dr. W. P. Roberts, of Jonesville, Wis., next spoke of pioneer work that was being done in his State for the prevention of tuberculosis.

Dr. Mayfield, of St. Louis, Mo., introduced a resolution at this point, which was referred to the committee on resolutions.

At this juncture the Secretary was authorized to read a letter from Dr. S. R. Burroughs, of Buffalo, Texas, one of the Vice-Presidents, and a delegate to the Congress, which referred forcibly to the work to be accomplished by this organization, and presenting his regrets at being unable to attend. The meeting adjourned until the next morning.

THIRD DAY, OCTOBER 5.

Dr. E. J. Barrick, President of the Congress, on being called upon, spoke in detail concerning the method of treatment in the municipal sanatoria in the different municipalities of Canada, and how the money for same was being obtained, speaking somewhat in detail concerning the methods of construction and plans for obtaining money and the successful management of these cases. He did not believe in voting free sanitariums except in rare instances for the very dependent. He thought every one who made as much as a dollar or a dollar and a half per day could pay something for treatment in such an institution.

Dr. De Witt, of Nova Scotia, also referred to the value of municipal sanatoria in Canada, and agreed with Dr. Barrick in all he had said.

Dr. J. L. Jacobsen, of Havana, Cuba, stated that he had recommended, in a paper before the Academy of Science in 1894, in Havana, the establishment of a sanatorium in La Sierra, a most healthy location in the province of Santa Clara, about eight hundred meters above the sea level. The Cuban League against tuberculosis recommended at several of its sessions the creation of popular sanatoria, and that the government had bought about one hundred acres of land at a cost of $17,000 for that purpose. The House of Representatives of Cuba voted a loan of $150,000, and before this money becomes available it is necessary that same be passed by the Senate. The speaker thought that favorable action would be obtained in that body.

Dr. Matto, of Lima, Peru, spoke eloquently concerning the altitude and climate of Peru as being most suitable for the treatment of tuberculosis. Owing to the fact that the speaker used the French language, the Secretary was not able to get full notes.

Dr. Ross, of Colorado, also spoke concerning the question of the prevention of tuberculosis, and discussed the value of fresh air, not only in the care, but in the treatment of the disease.

Dr. M. M. Smith, of Texas, spoke freely of the value of sanatoria in the treatment of the disease, as obtained by a visitation to nearly all of the sanatoria in the United States, and particularly the government sanatorium at Fort Bayard, New Mexico, and in his firm belief in that line of treatment.

Dr. Finarty, of Iowa, spoke and said he did not believe in legal restriction in the prevention of the disease.

Dr. Q. Kohnke, of Louisiana, President of the Board of Health of New Orleans, replied to those who did not believe in the germ theory and communicability of the disease, speaking at some length of the certainty of these theories as same were accepted by almost all authorities.

Dr. Mayfield, of Missouri, introduced Mr. Edison, President of the Fraternal World, which has a membership of about six millions in the United States. He was invited to address the Convention. He stated that his people expected to raise a large amount of money, and in all likelihood $1,000,000 for the establishment of sanatoria for the treatment of consumption, for the different orders, in a suitable climate. He believed in the active efforts to prevent the spread of tuberculosis, among the people at large. A vote of thanks was tendered Mr. Edison for his address, and the Fraternal World for its co-operation and assistance to the Convention in this philanthropic work. Mr. Edison pledged for the Fraternal World its support and co-operation.

AFTERNOON SESSION.

The report of the nominating committee was presented, and same was accepted, and, by motion, the Secretary of the Congress was instructed to cast the ballot of the Convention for each officer presented by the nominating committee. The motion prevailed, and the following officers were duly elected:

President, Dr. F. E. Daniel, Austin, Texas.
First Vice-President, Dr. J. H. Kellogg, Battle Creek, Mich.
Second Vice-President, Dr. Chas. Wood Fassett, St. Joseph, Mo.
Third Vice-President, Dr. John Ferguson, Toronto, Canada.

Fourth Vice-President, Hon. L. O. Nelson, Edwardsville, Ill.
Fifth Vice-President, Hon. Pat Dyer, St. Louis.
Secretary, Dr. M. M. Smith, Austin, Texas.
Treasurer, Hon. Clark Bell, New York,* N. Y.
Chairman of Council, Dr. P. O. Hanford, Colorado Springs, Colo.

The nominating committee also recommended the election of the usual large number of honorary presidents and vice-presidents, and the vice-presidents at large to cover all of the States and Provinces of the Western Hemisphere.

At this juncture Mr. Fuller, of New York City, representing the National Association of Civil Engineers, which was holding its annual convention at the World's Fair, was introduced, as a resolution in the form of an invitation had been extended by this Congress to that association. Mr. Fuller spoke concerning the great work being done by this association along the line of the prevention of disease, particularly concerning street cleaning and the proper disposal of sewage in the large cities.

Mr. Weston, of Boston, Mass., another representative from this Engineers' Congress, also spoke of the work being done along sanitary lines by his association. He referred particularly to the most excellent work done at the Stony View sanitarium in his city. He returned thanks to the Congress for their cordial invitation, and pledged the co-operation of their association in this work.

At this juncture the Hon. Clark Bell introduced a resolution thanking the sanitary engineers for the proffer of their aid and hearty co-operation at all times. Motion was adopted.

President E. J. Barrick, joined by Judge Bell, believed it would be of great value to have the assistance of the manufacturers and business men in order to accomplish the best results, particularly in an educational way, urging the appointment of a committee to assist in getting their co-operation and financial assistance in carrying out this excellent work.

Dr. J. H. Kellogg, of Battle Creek, Mich., stated that during the sitting of this Convention he had been in conversation with some leading philanthropists of the country—men whose means would allow them to contribute liberally in the support of the views set forth by this Congress for the prevention of tuberculosis in its various forms—and he felt he was in a position to pledge their financial assistance in carrying out this educational work. He asked that the Hon. John Patterson, of Dayton, Ohio, and the Hon. Horace Fletcher, of Venice, Italy, be elected to membership in this Association, which was done, and in addition they were elected honorary vice-presidents.

Nominations for chairman of committees were made. Dr. J. H. Kellogg, of Battle Creek, Mich., was made chairman of the sanatorium and educational committee, with authority to appoint the other members of his committee. Dr. P. O. Hanford, of Colorado

*To serve after January 1, 1905, only until his successor is chosen.

Springs, Colo., was appointed chairman of committee on legislation, with authority to appoint the other members of his committee. Dr. C. E. Cantrell, of Greenville, Texas, was made chairman of the committee relating to tuberculosis in venereal diseases, with authority to appoint the other members of his committee.

The report of committees on resolutions was presented, and the following resolutions were recommended and adopted by the Convention:

RESOLUTIONS ADOPTED BY THE CONGRESS.

Dr. F. E. Daniel, of Texas, offered the following preamble and resolutions:

WHEREAS, The public health is the paramount interest of every State and nation, and upon it depend in a measure all other interests; and

WHEREAS, In American countries it is less safeguarded than any interests public, private, personal, or property; and

WHEREAS, Consumption is the greatest menace and destructive agent to the public health, its mortality, according to the last United States census report, being over 400 a day; and

WHEREAS, The cause or causes of this disease and its methods of dissemination are well understood by medical and sanitary scholars; and

WHEREAS, It is known to be an infectious and communicable disease the prevention of which is within the power and scope of sanitary science; and

WHEREAS, It requires the authority of law to institute and enforce the necessary and proper measures of prevention; be it

*Resolved,* That it is the sense of the American International Congress on Tuberculosis, now in session at St. Louis, Mo., that it is the imperative duty of all civilized governments to take immediate action for the arrest of the spread of this scourge, and, as far as lies within the power of sanitary science and human endeavor, to eradicate it; and, further, that it is the sense of this Congress that every government should appoint a commissioner of the public health with a seat in the Cabinet, and give adequate authority and means to accomplish the desired ends in suppressing tuberculosis; and, further, that each State and Territory of the United States, where there exists a State Board of Health,—and in those States where there is no board of health there should be created a board composed of the ablest sanitarians,—authority should be given to such boards to formulate and enforce a code of regulations for the prevention of the ravages of this fatal scourge.

RESOLUTION OFFERED BY DR. E. J. BARRICK, OF TORONTO.

*Resolved,* That it is the duty of government authorities to promote the establishment and maintenance of State and municipal sanitaria in which tubercular patients may be isolated from their relatives and the public, and where they may be placed under suitable conditions for the cure or arrest of the disease.

RESOLUTION OFFERED BY DR. J. H. KELLOGG, OF BATTLE CREEK, MICH.

RESOLVED, That a committee be appointed to study, in a thorough-going way, the following questions:

1. What are the best plans for the construction and management of hospitals, sanitarium buildings, cottages, tents, and other structures for the accommodation of tubercular patients?

2. What should be the diet of the tubercular patient, especially with reference to the kind and quantity of proteids, fats, and carbohydrates?

3. What accessory, physical and hygienic, measures may be properly employed, such as sun-bathing, the electric light as a substitute for sunlight, cold bathing, exercise, special gymnastics, etc.?

4. What simple rules of hygiene, applicable in the home, may be commended as preventive of tuberculosis? and

*Resolved,* That the chair appoint a committee whose duty it shall be to collect from all available sources accurate data in relation to the several questions above named, and to digest and formulate the same into a series of recommendations for general circulation; and

*Resolved,* That it is the duty of health boards, health officers, and all sanitary authorities to report cases of this disease, at least all cases of pulmonary tuberculosis, with other infectious disorders, and to observe the same care as regards disinfection of the premises occupied by persons suffering from this malady, as after measles, or any other contagious disease; and

*Resolved,* That to facilitate the early diagnosis of this disease, it is the duty of every State and municipality to provide laboratory facilities sufficient for its needs for the microscopic and bacteriologic examination of sputa; and

*Resolved,* That systematic efforts should be made for the education of the public in relation to the communicability and curability of tuberculosis through the circulation of carefully prepared leaflets and other literature, the holding of schools of health or health conventions, the organization of local and State societies for the suppression of tuberculosis, special lectures before fraternal associations, clubs, college students, chautauquas, associations of teachers, clergymen, and other bodies of professional men; and

*Resolved,* That in our hospitals for the insane and other hospitals and asylums, as well as prisons and alms houses, provision should be made for the isolation of tubercular persons.

RESOLUTION OFFERED BY DR. DENSMORE LEWIS.

*Resolved,* That a special committee be appointed to investigate the relation between venereal disease and general or local tuberculosis.

RESOLUTION OFFERED BY DR. P. O. HANFORD, OF COLORADO SPRINGS.

*Resolved,* That a national legislative committee should be appointed to receive the memorials and resolutions passed by the committee on resolutions of this Congress, and to take such measures as to have them properly presented in the Congress of the United States and the governments of the foreign countries represented in this Congress;

That they be empowered further to confer with the legal representatives of each State municipality, and even local boards of health, to the advancement of local legislation against the spread of tuberculosis;

That they be empowered to draft, compile or formulate a copy of a general law, and urge each State to enact the same under the authority of this Congress.

The suggestion is made that this committee be composed of professional men representing those professions whose judgment will advance the aëration, lighting and sanitation of dwellings, pathology, etc.; and that an honorary board be formed to act in conjunction with committees from business men's associations, fraternal associations, and others who may aid in this great work.

Further that this committee appoint in each State one person to act in the formation of like committees within the State.

RESOLUTION BY J. H. KELLOGG, BATTLE CREEK, MICH.

*Resolved,* That the chair be authorized to appoint a committee of four persons who shall constitute a committee on publicity, and who shall be empowered to enlarge their number by enlisting the assistance and co-operation of prominent educators, manufacturers, philanthropists, and others who may be able in various ways to assist in carrying on a strong educational campaign against tuberculosis.

RESOLUTION OFFERED BY DR. MAYFIELD, OF ST. LOUIS.

*Resolved,* That we most heartily concur in the suggestion of the National Fraternal Congress, recently in session in this city, representing more than five million persons, that hospital cars should be provided by the leading railroad lines connecting with climatic resorts frequented by tubercular patients; and that we recommend the efforts as one in which all organizations and associations of men and women should be interested.

Dr. F. E. Daniel, of Austin, Texas, the newly-elected President, was introduced, and spoke as follows:

*Mr. Chairman and Gentlemen:*

The distinguished honor it has been your pleasure to confer upon me is as unexpected as it is undeserved. Your selection should have fallen upon one abler and better known than myself, and a representative from one of the older States. It should have been conferred upon some distinguished man who, by his work, is more fully identified with the subject. There are many here better fitted for it and better entitled to the honor than myself; there are those who have the time and the special fitness and the means to bestow upon the great work we have undertaken. I seriously mistrust my ability to meet your expectations. But on my interest, nay, inthusiasm in the cause there is no discount. In an humble way and a limited sphere I have done my best for the cause. I have sown seeds, most of which have fallen, I fear, in stony places, and I see few results. It is a matter of pride with me, however, to believe that some have fallen in good soil and have borne fruit. It is no spirit of vanity, but one of pardonable pride, to say that I believe I was the first one to advocate and urge a radical change in the construction and equipment of sleeping cars. Fifteen years ago I began the campaign, and have pushed it constantly by tongue and pen. The sleeping cars in general use are overheated, badly ventilated and are equipped with furnishings that harbor the germs of disease, and they can not be entirely disinfected. One literally takes his life in his hand when he sleeps in one of them. I advocated an aseptic car—roomy, well ventilated and equipped with rattan and linen, and rubber, instead of woolens, plush and fancy carvings. You may imagine my delight when on starting to St. Louis I found myself in my ideal car, my dream, on that model and progressive railroad, the "Texas Road," the great I. & G. N., of which all Texas is justly proud. This road, upon the advice of its chief surgeon, Dr. W. G. Jameson, who is today present as a delegate to this Congress, was the first railroad in the world, so far as I know, to disinfect sleeping cars by formaldehyde, and Texas has the proud distinction of being the first State to pass a law requiring it. It is not vanity but a justifiable pride that claims for my advocacy a part, at least, of the credit for that reform.

In accepting the honor you have conferred upon me—and the burden—I will, in the future, as in the past, do all in my power to carry forward the great and glorious work of sanitary reform. Preventive medicine with this Congress, aims primarily at that fell destroyer of human lives—consumption. We are confronted with a vast problem and have undertaken a Herculean task. We are but a handful, it is true, but in this assemblage of earnest workers, lay and medical sanitarians of recognized ability and renown, what potentiality resides and what achievements may result, is beyond conjecture. The pilgrim fathers were but a handful, but they reclaimed this great continent from the savages. The pioneers who pushed across the waste places—the deadly deserts and the formidable Rockies were but a handful, but they won the great West—the future home of teeming millions, and gave to our energies and products an outlet on the great Pacific. We are but a handful, but, undismayed, we have entered upon the mighty work for humanity and race integrity, encouraged rather, by the record in recent years, of sanitary science which has banished smallpox, banished yellow fever, disarmed of their terrors diphtheria and even bubonic plague. We are encouragd, moreover, by the alliance already made, this first meeting, with the National Association of Civil Engineers. We are honored this afternoon by the presence of their representatives in the person of their President and Secretary, who came to assure us of their co-operation and support. Already, too, we have attracted and won to this Congress the great Fraternal League of America, a consolidated body of all the fraternities, whose membership now reaches five and a half millions, and who have been fighting consumption single-handed. They have joined forces with this Congress, and we welcome them gladly. They will co-operate with us and will be represented on our next assembly. And last, and the most gratifying of all, we have effected an alliance with and secured the support and co-operation of the Woman's League, fittingly represented on this floor in the charming person of their delegates. God bless them all!

"They talk about a woman's sphere
As though it had a limit;
There's not a place in earth or heaven,
There's not a task to mankind given,
There's not a blessing or a woe,
There's not a whisper, yes or no,
There's not a life, there not a birth,
That has a feather's weight of worth—
Without a woman in it."

Thus, as in world-building, a nucleus, a center of attraction, has drawn to it independent and detached particles and forces, and acquired increased momentum. Thus will the work of this Congress go on until it will fully unify all the leagues and other organizations for war against the giant, consumption, and like a mighty river it will go on and on with a rush and resistless force till the great humanitarian end is reached, till consumption shall be circumscribed and inhibited in its deadly work. It must be rendered powerless for harm. Not only is it the deplorable loss of life every year that is so appalling, but where one dies there are perhaps a dozen sick and incapacitated, yet who transmit at least the predisposition, a weakened vitality to coming generations. This threatens our race integrity. The public health is the foundation upon which depend the strength, efficiency, progress, even the existence of a State or nation. Given strong, healthy units and we have strong, aggressive and progressive civilization. An enfeebled nation never carried the blessings of civilization to a benighted world or held its place in the struggle for existence or supremacy.

But this brings up a consideration of that deeper problem—not the arrest of the spread of the infection, but the eradication of the disease. This problem lies at the root of our social fabric; consumption is a disease of civilization, and is inherent in our lives and industries. It is a house disease, and flourishes because of unsanitary dwellings and factories, and modes of travel, of living, and labor. And here is where the engineers come in; here is where their labors touch ours. They are the most powerful factors in that reform which can alone diminish, not to say eradicate, the disease. They have to do with the construction of buildings for living and for labor; with sewers and waterworks—those great purifiers; with heating and ventilation; with plumbing and drainage, and with street cleaning, hence with dust, the great and terrible distributor of infection. The government should have the supervision under a capable and honest engineer—of the construction of all buildings, public and private, for residence or work, and especially of those of the laboring classes. No soulless landlord should be permitted to crowd tenement houses on insufficient ground and deprive the poor of God's sunlight and air. An abundance of both, with plenty of pure water, is a God-given right. Crowding of whole families into one small apartment, where they eat, sleep and work in a suffocating dark air, should be prohibited, for there is the hot-bed of the disease, there the monster, tuberculosis, has his lair. Already much has been done in this direction in New York, and the death rate of consumption has materially decreased.

It is comparatively easy to prevent the spread of the infection under favorable circumstances. The poison is in the sputa, and, if this be destroyed, the consumptive is powerless for harm. It is a mistake to suppose that the disease is contagious; it is in no sense contagious, but is infectious. It can not be acquired simply by contact, and it is an open question still, if it can be transmitted by other means than by the introduction into the lungs of the tubercle bacilli, most frequently in dust. And, even then, there must be a suitable *nidus,* or it will not germinate. Healthy persons have, in many instances, the living germs in the mouth and throat; but still the consumptive should in a measure be separated from the other members of the family, for breathing his expired air *may* communicate the disease; hence the danger of crowding. Sunlight and air, while a vital necessity, will not disinfect a room.

But it takes authority and means to enforce even the simplest precautions, and somebody must think and act for the toiling millions—the understratum of society—who occupy the slums and other unsanitary places. They can not be reached by any "Campaign of Education." You can not reach them with your literature, and, if you could, they would not read or understand it, and they are powerless to act. The government should be paternal, and take care of them, if not for their own sake, for the protection of the public and in the interest of race integrity.

And here let me sound a note of warning. In all reforms there is apt to be a reaction, and, in striking at one evil, we are apt to create another. We are apt to go to extremes. Witness the French Revolution as an illustration. When, revolting against centuries of oppression and wrong, the people rose in rebellion; when the Jacobins had overthrown the conservatives, the Girondins, when the French King and Queen had been led to the scaffold, when the aristocracy had been exterminated, mad with the lust of blood, the revolutionists turned upon each other, and a reign of terror followed which shocked and paralyzed the civilized world. Let us be careful that in our crusade against the disease, consumption, we do not make war upon and wrong and outrage the consumptive. He is not a subject for quarantine. It is not a quarantinable disease. He is not, like a leper, unclean, unclean, and to be shunned like a pestilence. If the poison he expectorates and the air vitiated by his breath be avoided, he is as powerless for harm as this table. He should not be shut out from your States. In Colorado and in California and in Texas many, very many of the best citizens went there either invalids from consumption, or for the health of some member of the family. The Commission of Immigration in June, 1902, on an opinion of the then Attorney General, decided that consumption comes within the scope of the Federal law excluding immigrants suffering with "a contagious disease dangerous to the public health," and under this ruling an immigrant was actually separated from his family and not allowed to land, but was sent back whence he came.* This is a great wrong, an injustice, and such exclusion by any State will work wrong and injury and inhumanity. California should welcome the consumptives, but not by quarantine.** They should be segregated, if bed-ridden, and given the benefit of the pure air and sun and wholesome fruits of that God-favored clime.

I have spoken longer than I intended. I am full of the subject, and "out of the fullness of the heart the mouth speaketh." I take up the burden you have placed on my shoulders, and enter into the great work with hope and encouragement. I ask you to hold up my hands even to the going down of the sun, and the battle for humanity will be won.

To the attainment of the great ends for which this Congress was created, I dedicate the remaining years of my life, and pledge my most earnest endeavors.

Dr. J. H. Kellogg, of Michigan, First Vice-President, was called upon, and he pledged the Congress his co-operation and assistance at all times; furthermore, the ample funds that he would attain through his committee from the philanthropists to carry out this educational work in its various channels.

Dr. M. M. Smith, Secretary, being called, in a few words referred to the importance of the work before us, and spoke of the necessity of adopting the most thorough business methods in the conduct of the Association in all of its work, and believed, with the money promised, they would be able to carry out the recommendation.

Dr. Kohnke, of Louisiana, a member of the council, was called upon for a speech, and made many valuable suggestions as to the course to be pursued, and as to the best means to accomplish results in this work.

---

*Since this address was delivered a distinguished judge from Tahitti, suffering with consumption, was on his way to Europe, via San Francisco and New York. The California authorities refused him permission to land and cross the continent to New York.—Ed.

**California delegates advised exclusion of consumptives, or putting them in State lazarettos.—Ed.

Dr. David Matto, of Lima, Peru, expressed appreciation on behalf of the Latin delegation to the Congress, and pledged them their united support in this work in the future.

Dr. Pratto, of San Salvador, spoke eloquently concerning the work, and wished the Congress success.

Dr. Jennie DaCollum, of Davenport, Iowa, in a few choice words referred to the work being done in her city for the prevention of tuberculosis.

Dr. N. K. Foster, of California, was next called for, and referred to the large number of charity cases shipped into their country from different parts of the world, and who were led to believe that all that was necessary for them to do to recover was to breathe that air. He spoke of the great hardship, not only to the people, but to the patient. He advised conservatism in sending patients to a foreign land.

Dr. P. O. Hanford, of Colorado, chairman of the legislative committee, spoke of the value of police regulations concerning the suppression of disease, and believed by active efforts on the part of the legislative committee representing this Congress that much in the way of legislation could be obtained throughout the land.

Dr. Annie G. Lyle, of San Francisco, Cal., advocated harmoniout and earnest work in this Congress, and spoke of the large amount of good which might be obtained if they would work along educational lines with that determination and enthusiasm necessary to accomplish this end. Dr. Lyle pledged the hearty cooperation of the Pacific Slope in this great work.

At this juncture a resolution of thanks was introduced to the United States government, the Louisiana Purchase Exposition, the governors of the various States and Provinces, and the local committee and members for their co-operation and assistance in making this meeting a success.

Dr. F. E. Daniel, the newly-elected President, arose and asked the privilege of saying a few words. He paid a high tribute to the great work done in this line by the Hon. Clark Bell, of New York, and by the retiring President, Dr. E. J. Barrick, of Toronto, Canada. After which Judge Bell arose and tendered his sincere thanks to Dr. Daniel for his remarks. He assured the Congress these remarks were not altogether deserved, but that his heart and soul was in the work, though retired from active duties as an officer of the Congress. He would give such aid and assistance as were in his power in his declining years. After this the Congress adjourned to meet at such time and place as the executive committee might determine, which, in all likelihool, will be three or four days before the meeting of the American Medical Association, in whatever place that meeting will be held in 1906.

The old officers and committees of the American Congress on Tuberculosis as now in existence hold over to January 1, 1905, and to the executive board now in office is entrusted the closing up of the work of the sessions of 1903 and 1904, and the selection of delegates to home and foreign societies and congresses in any wise relating to tuberculosis.

